# MiR-652 inhibits acidic microenvironment-induced epithelial-mesenchymal transition of pancreatic cancer cells by targeting ZEB1

**DOI:** 10.18632/oncotarget.5350

**Published:** 2015-10-19

**Authors:** Shichang Deng, Xiang Li, Yi Niu, Shuai Zhu, Yan Jin, Shijiang Deng, Jingyuan Chen, Yang Liu, Chi He, Tao Yin, Zhiyong Yang, Jing Tao, Jiongxin Xiong, Heshui Wu, Chunyou Wang, Gang Zhao

**Affiliations:** ^1^ Pancreatic Disease Institute, Union Hospital, Tongji Medical College, Huazhong University of Science and Technology, Wuhan, 430022, China; ^2^ Gastrointestinal Surgery, Union Hospital West Campus, Tongji Medical College, Huazhong University of Science and Technology, Wuhan, 430056, China

**Keywords:** miR-652, ZEB1, epithelial-mesenchymal transition, acidic microenvironment, pancreatic cancer

## Abstract

Recent evidences suggest that the acidic microenvironment might facilitate epithelial mesenchymal transition (EMT) of tumor cells, while the effects of acidity on EMT of pancreatic cancer (PC) remain undefined. The present study demonstrated that acidity suppressed miR-652 expression, which further promoted EMT process by absenting inhibition on the transcriptional factor ZEB1 expression. At first, we found that acidity remarkably enhanced invasion ability of PC cells accompanying with increased mesenchymal and decreased epithelial markers. Meanwhile, miRNAs-microarray showed that miR-652, the potential regulator of ZEB1, was distinctly decreased in acidity-treated PC cells. Furthermore, restoration of miR-652 reversed acidity-induced EMT by inhibiting ZEB1 expression, while miR-652 inhibitor induced EMT in normal PC cells through promoting ZEB1 expression. Nevertheless, knockdown of ZEB1 significantly suppressed acidity-induced EMT in PC cells, but ZEB1 overexpression rescued the EMT which was inhibited by miR-652 overexpression. The *in vivo* results showed that the tumor growth and liver metastasis were remarkably retarded by both miR-652 overexpression and ZEB1 knockdown. The clinical samples further revealed that miR-652 was decreased in PC tissues and antagonistically correlated with ZEB1 expression, associating with late tumor stage, lymphatic invasion and metastasis. In conclusion, our study indicated a novel acidity/miR-652/ZEB1/EMT axis in the tumorigenesis of PC.

## INTRODUCTION

Emerging evidences have indicated extracellular acidity is an environmental stimulus that may play a key role in tumorigenesis [1–3]. Resulted from high glycolytic rate and lactic acid production coinciding with an insufficient drainage by convective and/or diffusive transport, H^+^ ions accumulate in the respective tissues of cancer [4]. As a consequence, both *in vitro* cell culture studies and *in situ* tumor spectroscopic studies utilizing the ^31^P isotope have reported that tumor cells have acidic interstitial extracellular pH (pHe) values compared to normal tissues (6.2–6.9 vs 7.3–7.4), especially in bulky and/or low-flow tumors [5, 6]. Evidences indicate extracellular acidity can decrease the radiosensitivity of mammalian cells, modulate the cytotoxicity of certain anticancer drugs, as well as promote tumor invasion and metastasis [7–9]. Similar to the other solid tumors, pancreatic cancer is also characterized by acidic regions resulting from a switch of cellular metabolism to glycolysis with accumulation of lactic acid in the extracellular space [10, 11]. Moreover, the remarkable extracellular acidity is augmented by the characterized hypovascular of pancreatic cancer [12, 13]. It draws our intensive interesting whether the vigorous acidic extracellular microenvironment also contributes to the development and progression of pancreatic cancer.

Epithelial to mesenchymal transition (EMT) was firstly identified as a crucial differentiation and morphogenetic physiological process by facilitating cell movements and generation of new tissue types during embryogenesis [14]. Accumulating evidences suggested the EMT process plays important roles in tumor progression, including pancreatic cancer [15–18]. Our previous study also revealed that both chronic pancreatitis and pancreatic cancer demonstrated active EMT process, which indicated the pivotal role of EMT in tumorigenesis of pancreatic cancer [19]. Since recent researches demonstrated that the acidic microenvironment could induce EMT progression in lung carcinoma and melanoma cells [20, 21], and the induction of EMT in tumors appeared to be highly tissue- and cell type-specific [22], it was required for us to explore whether acidic microenvironment could contribute to EMT activation and further involve in tumorigenesis of pancreatic cancer.

MicroRNAs (miRNAs) are small non-coding RNAs that bind mRNAs of potentially hundreds of genes at the 3′ UTR, resulting in degradation or inhibition of the target mRNAs. Aberrant expression of miRNAs contributes to carcinogenesis by promoting the proto-oncogenes or inhibiting the tumor suppressor genes, including pancreatic cancer. [23–25]. Recently, a wealth of evidences from our laboratory and others highlighted the roles of miRNAs in EMT process [19, 24–26]. Moreover, miRNAs, acting as the crucial stress-responsive mediator, are involved in the signal transduction in response to stress including hypoxia or inflammation stimulus [19, 27–29]. Therefore, we speculated that the responsively dysregulated miRNAs may participate in signal transduction between tumor acidic microenvironment and EMT. The present study applied miRNAs-microarray to explore the miRNAs panel involved in the acidity-induced EMT of pancreatic cancer cells. Among those dysregulating miRNAs in acidity, miR-652 was an attractive candidate functioning in acidic microenvironment with significant downregulation. Importantly, ZEB1, the transcription factor for EMT exerting critical roles in malignant progression of cancers including pancreatic cancer [30, 31], might be potential target of miR-652 basing on bioinformatics prediction. Therefore, we further detect whether miR-652 exerts critical roles in acidity-induced EMT of pancreatic cancer cells.

In this study, we established the first comprehensive miRNAs expression profiles in pancreatic cancer cells treated in acidic condition, aiming to identify the functional miRNAs which are potential regulators for acidity-induced EMT in pancreatic cancer and further revealed their direct targets. Moreover, it would provide novel insights into an acidity-induced EMT axis in progression of pancreatic cancer, which offers new candidate targets to be exploited for diagnostic and therapeutic strategies.

## RESULTS

### Extracellular acidity induces EMT in pancreatic cancer cells

**Figure 1:**
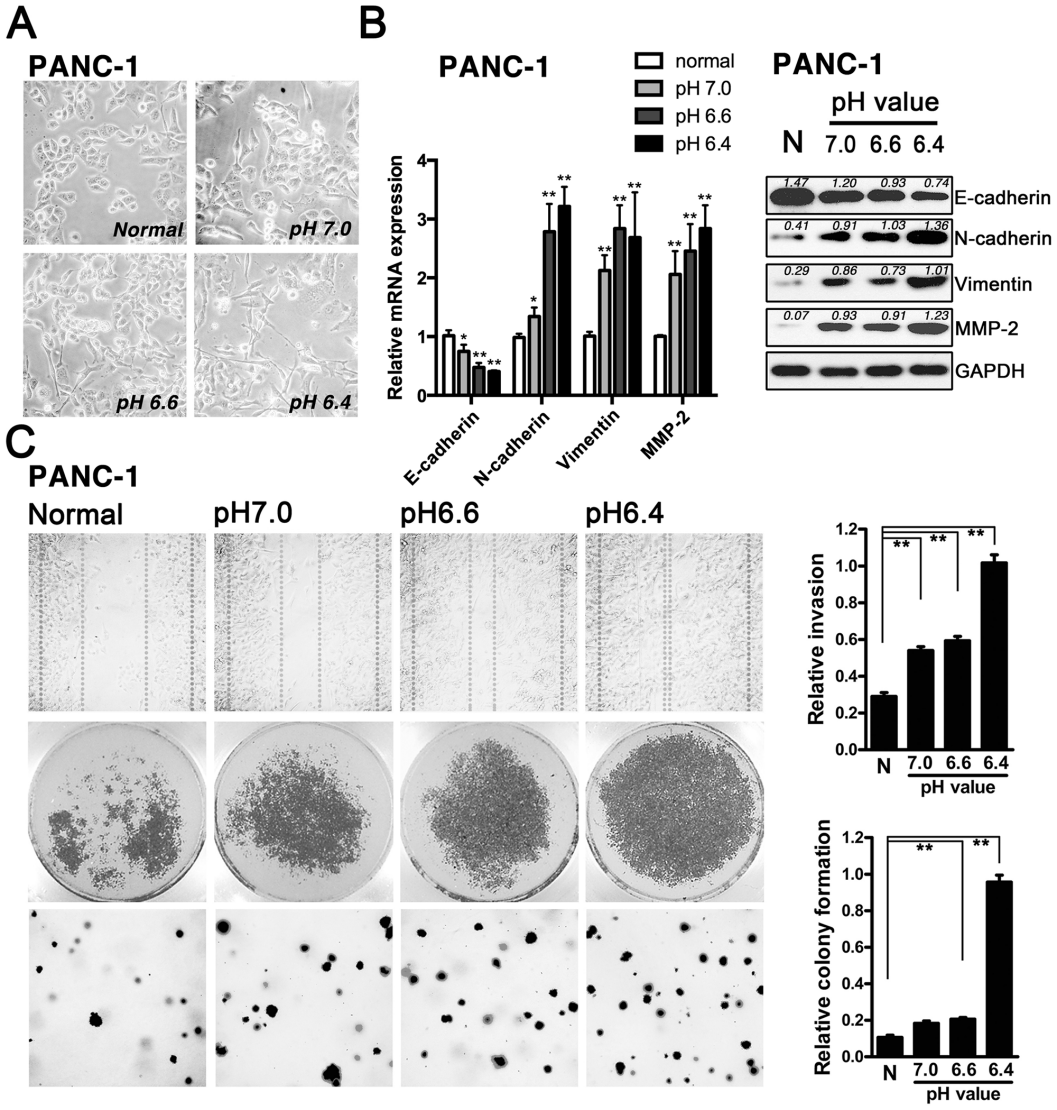
Acidity potentiated EMT in pancreatic cancer cells **A.** PANC-1 cells were incubated in normal (pH7.4) and acidic medium (pH7.0, pH6.6, pH6.4) for 72 h and the monolayer morphology was photographed at X20 magnification. **B.** qRT-PCR (*Left panel*) and Western blot (*Right panel*)analysis of epithelial markers (E-cadherin), mesenchymal markers (N-cadherin, Vimentin and MMP-2) protein levels in PANC-1 cells kept at normal (pH7.4) and acidic medium as indicated up to 72 h. **C.** Acidic pH increased cell motility (*Left panel*: *Upper*: wound healing assay, X20), invasion ability (*Left panel*: *Middle*: cell invasion assay) and colony formation ability (*Left panel*: *Lower*: soft agar colony formation assay, X20) of PANC-1 cells. Quantification of cell invasion assay (*Right panel*: *Upper*) and soft agar colony formation assay (*Right panel*: *Lower*). The graphs represent data from three separate experiments. Values are significant at **P < 0.01 and *P < 0.05 as indicated.

In order to address whether acidic microenvironment potentiated EMT process, pancreatic cancer cell lines (AsPC-1, BxPC-3, PANC-1, MIAPaCa-2, SW1990) were incubated under normal (pH 7.4) and different acidic conditions (pH 7.0; pH 6.6; pH 6.4) up to 72 h. Typically showed in PANC-1 cells, the exposure to acidic microenvironment led cells undergo marked EMT-like transformation, evidenced by alterations of morphology from an epithelial (a polygonal shape and cobblestone-like sheets) to a more mensenchymal (loose cell contacts, scattered from cell cluster and acquired an elongated, fusiform morphology with dendritic processes) phenotype (Fig. 1A). In the meantime, the acid treated PANC-1 cells demonstrated loss of epithelial marker (E-cadherin) and gain of mesenchymal markers (N-cadherin, Vimentin and MMP-2) (Fig. 1B), as well as significant enhanced cell motility, invasion and colony formation ability (Fig. 1C). However, proliferation of PANC-1 was retarded in acidic medium (Fig. S1A), as well as that of other cell lines (Fig. S1B). Moreover, the similar results of acidity-induced EMT in AsPC-1, BxPC-3, MIAPaCa-2 and SW1990 were observed and shown in Fig. S2 and Fig. S3. Corresponding data of cell cycle exhibited acidity-induced G1-phase arrest in pancreatic cancer cells (PANC-1 and BxPC-3, Fig. S1C). Collectively, these data revealed that acidic microenvironment could induce EMT of pancreatic cancer cells (Table 1).

**Table 1:** The criteria for acidity-induced EMT-like phenotype of pancreatic cancer cell lines

Cell Lines	Epithelial Marker	Mesenchymal Marker	Cell Motility	Cell Invasion	Colony Formation
PANC-1-A	*Loss*	*Gain*	*Enhanced*	*Enhanced*	*Enhanced*
BxPC-3-A	*Loss*	*Gain*	*Enhanced*	*Enhanced*	*Enhanced*
AsPC-1-A	*Loss*	*Gain*	*Enhanced*	*Enhanced*	*Enhanced*
MIAPaCa-2-A	*Loss*	*Gain*	*Enhanced*	*Enhanced*	*Enhanced*
SW1990-A	*Loss*	*Gain*	*Enhanced*	*Enhanced*	*Enhanced*

Cell lines-A: pancreatic cancer cells that were incubated in pH 6.4 value medium for 72 h.

Epithelial Marker: E-cadherin was measured.

Mesenchymal Marker: N-cadherin, Vimentin and MMP-2 were measured.

### Extracellular acidity significantly downregulates miR-652 and upregulates its potential direct target ZEB1

**Figure 2:**
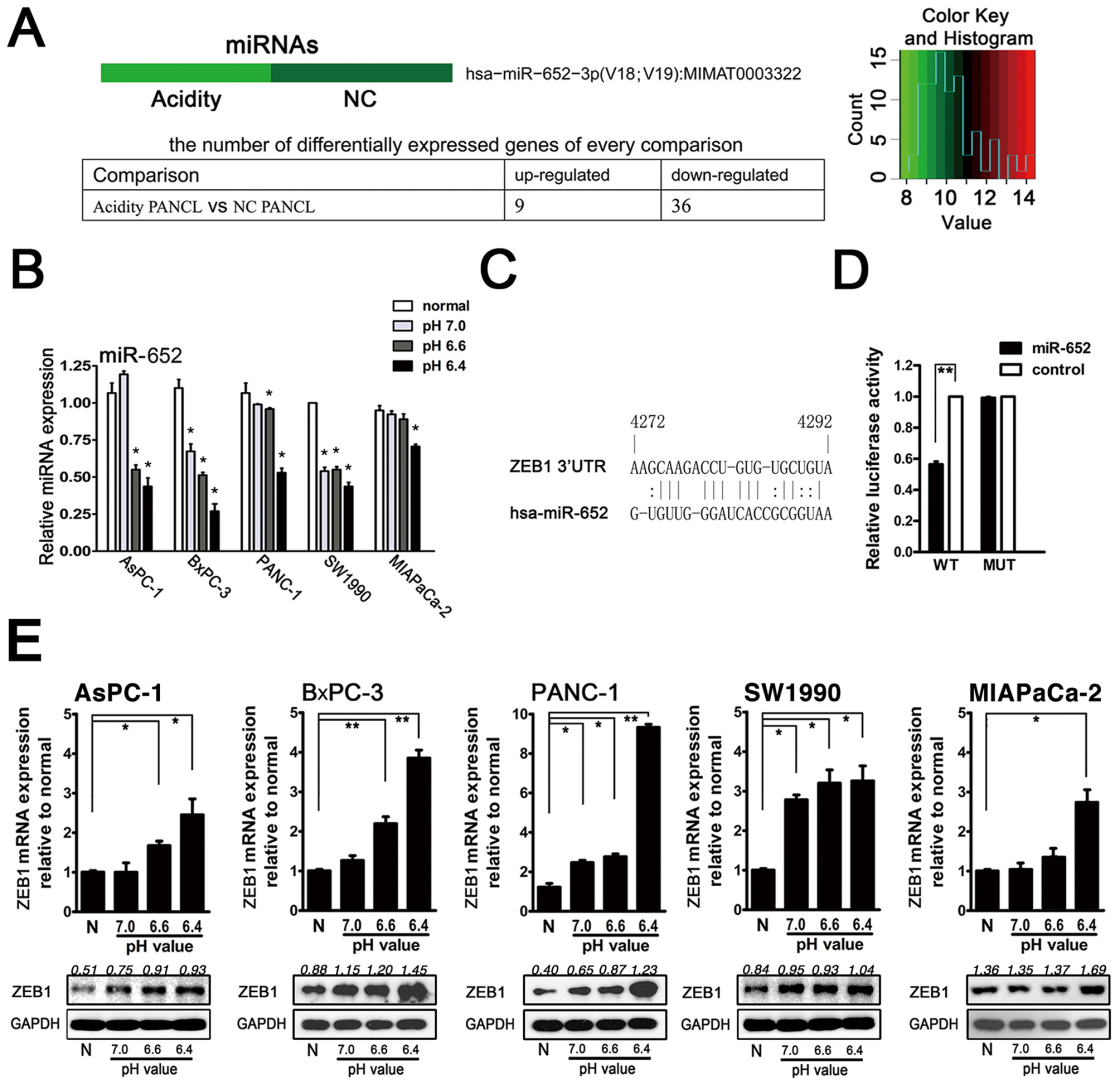
Acidity induced miR-652 downregulation and its direct target ZEB1 upregulation in pancreatic cancer cells **A.** MiRNAs-microarray analysis. Clustering analysis of Acidity *vs* NC (*Upper*). The number of differentially expressed microRNAs in acidity and NC in PANC-1 cells (*Lower*). Standard selection criteria to identify differentially expressed miRNAs are established at *P* < 0.05 and fold difference > 2. **B.** qRT-PCR analysis of miR-652 expression in AsPC-1, BxPC-3, PANC-1, SW1990 and MIAPaCa-2 cells cultured in normal and acidic medium as indicated for 72 h. **C.** Illustration of ZEB1 3′UTR as well as the seed sequence of miR-652 showing the predicted target region on the 3′UTR of ZEB1 mRNA. **D.** Luciferase reporter assay showed overexpression of 652 in PANC-1 cells led to decreased activity of ZEB1 3′UTR-luciferase construct respectively. **E.** qRT-PCR analysis of ZEB1 mRNA expression in AsPC-1, BxPC-3, PANC-1, SW1990 and MIAPaCa-2 cells kept in normal or acidic medium as indicated up to 72 h (*Upper*). Western blot analysis of ZEB1 protein levels in pancreatic cancer cells cultured at normal and acidic medium up to 72 h (*Lower*). U6 was used as the endogenous control for miR-652, and GAPDH for ZEB1. The graphs represent data from three separate experiments. Values are significant at **P < 0.01 and *P < 0.05 as indicated.

To further identify whether miRNAs involved in acidity-induced EMT process, miRNAs expression profiles of PANC-1 in acidic medium (pH = 6.4) were compared with that in normal medium (pH = 7.4) by miRNAs-microarray (RiboArray™ miDETECT™ Human Array 1 × 12K) carrying 2578 individual human miRNAs from the miRBase 20.0. The results showed that 9 miRNAs were upregulated and 36 miRNAs were downregulated more than 2 folds (Fig. 2A). Among those downregulated miRNAs, miR-652 was decreased as 2.39 folds. We further used qRT-PCR to identify the expression of miR-652 in pancreatic cancer cell lines after exposure to the acidic environment. The results showed the expression of miR-652 was decreased according to descent of the pH value in the pancreatic cancer cell lines (Fig. 2B). Based on the miRNAs target prediction bioinformatic software, miR-652 might be the regulator of ZEB1, which is the pivotal transcription factor for EMT (Fig. 2C). We further evaluated the ability of miR-652 to inhibit the ZEB1 expression via dual luciferase reporter assays. The pmiR-RB-Report™-ZEB1 (wild type ZEB1 3′UTR, WT) and pmiR-RB-Report™–Control (mutant type ZEB1 3′UTR, MUT) were purchased from RIBOBIO (Guangzhou, China). PANC-1 cells were transfected with miR-652 mimics. As shown in Fig. 2D, miR-652 inhibited the luciferase activity of the reporter with wild type 3′UTR of ZEB1, but not in mutant type 3′UTR of ZEB1. We further monitored alterations of ZEB1 expression under the different pH value conditions using qRT-PCR and western blot analysis. As the pH value decreased, ZEB1 expression significantly increased both in mRNA and protein levels in pancreatic cancer cell lines (Fig. 2E). These results indicated that the responsively decreased miR-652 might lead to the overexpression of ZEB1 in acidic microenvironment by derepressing the inhibition ZEB1 expression.

### Restoration of miR-652 reverses acidity-induced EMT in pancreatic cancer cells with ZEB1 downregulation

**Figure 3:**
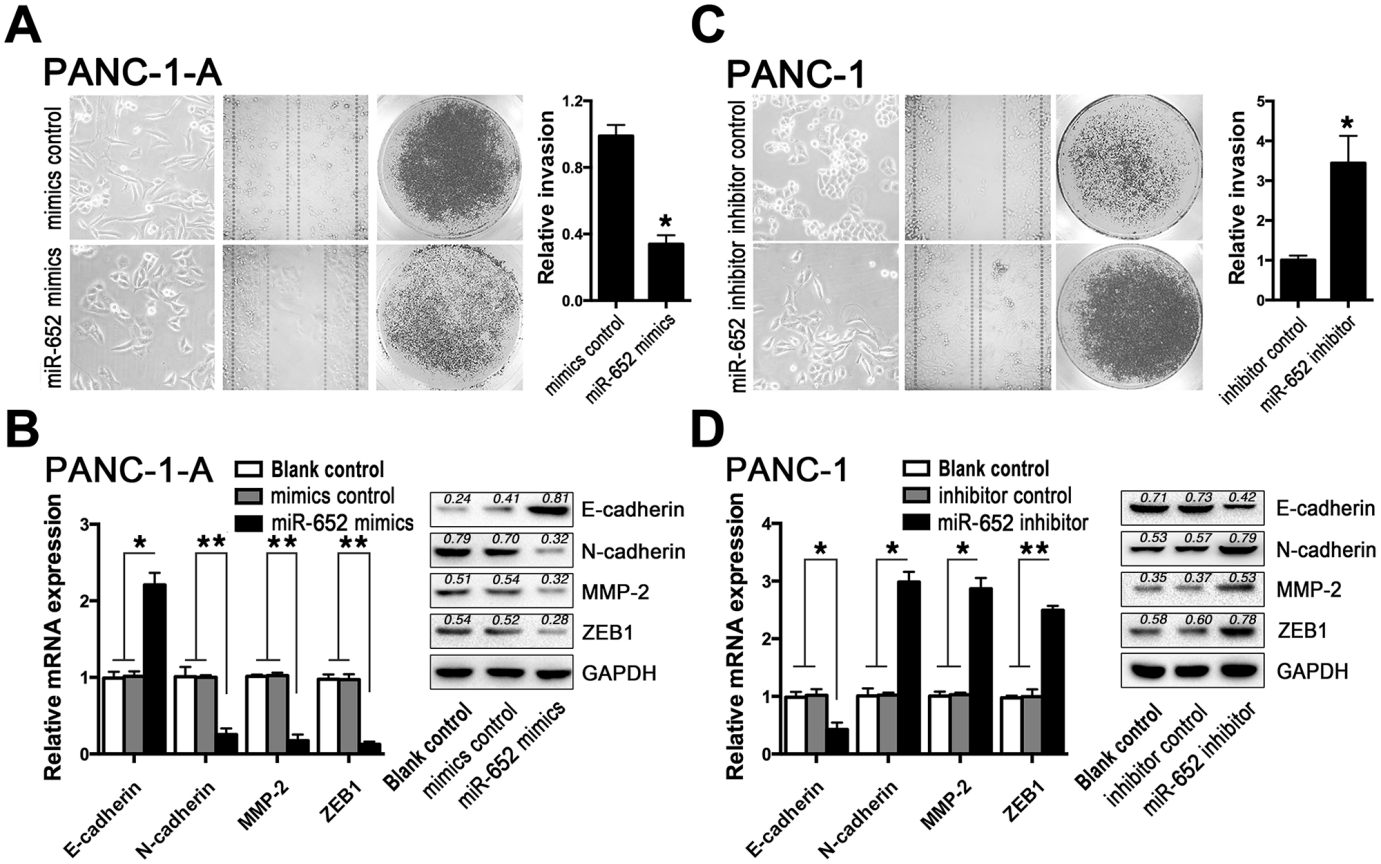
ZEB1 mediated miR-652 function in acidity-induced EMT **A.** Monolayer morphology, wound healing assay and cell invasion assay of PANC-1-A (PANC-1 incubated in pH 6.4 value medium for 72 h) after transfection of miR-652 mimics. Quantification of cell invasion assay is shown in histogram. **B.** qRT-PCR and corresponding western blot analysis of E-cadherin, N-cadherin, MMP-2 and ZEB1 in PANC-1-A after transfection of miR-652 mimics. **C.** Monolayer morphology, wound healing assay and cell invasion assay of PANC-1 cells after transfection of miR-652 inhibitor. Quantification of cell invasion assay is shown in histogram. **D.** qRT-PCR and corresponding western blot analysis of E-cadherin, N-cadherin, MMP-2 and ZEB1 in PANC-1 after transfection of miR-652 inhibitor. The graphs represent data from three separate experiments. Values are significant at **P < 0.01 and *P < 0.05 as indicated.

Since downregulation of miR-652 was associated with overexpressed ZEB1 in pancreatic cancer cells in extracellular acidity, we further explored whether restoration of miR-652 could reverse the extracellular acidity-induced EMT in pancreatic cancer cells. After restoration of miR-652, the acidity-treated pancreatic cancer cells (PANC-1-A and BxPC-3-A, incubated in pH 6.4 value medium for 72 h) demonstrated a dramatic MET (mesenchymal to epithelial transition) presenting with significant decreased cell motility, invasion ability (Fig. 3A), upregulated E-cadherin and downregulated N-cadherin, MMP-2 and ZEB1 (Fig. 3B). Notably, restoration of miR-652 in BxPC-3-A cells also showed the similar MET process as in BxPC-3-A cells (Fig. S4A and S4B).

### Inhibition of miR-652 simulates the acidity-induced EMT in pancreatic cancer cells by upregulating ZEB1

To further confirm the downregulated miR-652 in response to acidic microenvironment could promote EMT in pancreatic cancer cells, we inhibited the expression of miR-652 in pancreatic cancer cells under normal medium. Similar to what was observed in acidic conditions, transfection with miR-652 inhibitor induced mesenchymal-like morphological changes in PANC-1 cells with dramatically increased invasion ability and cell motility (Fig. 3C), as well as decreased E-cadherin and upregulation of N-cadherin, MMP-2, ZEB1 (Fig. 3D). Similarly, inhibition of miR-652 in BxPC-3 cells also showed the EMT process (Fig. S4C and S4D). Therefore, the above data intensively indicated that the responsively dysregulated miR-652 could upregulate ZEB1 to promote EMT of pancreatic cancer cells in extracellular acidity.

### Downregulation of ZEB1 reverses the extracellular acidity-induced EMT in pancreatic cancer cells

**Figure 4:**
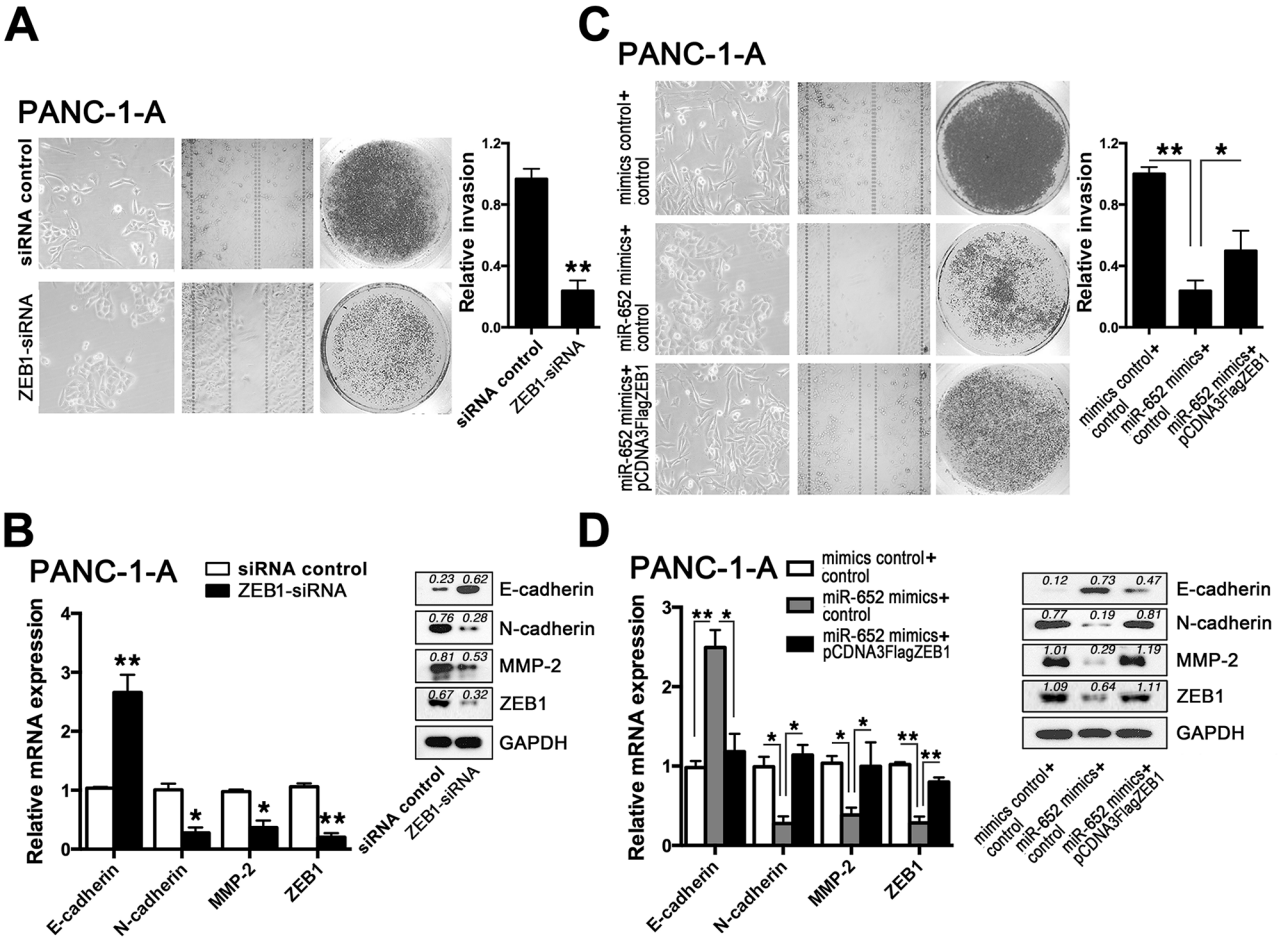
Downregulation of ZEB1 reversed the extracellular acidity-induced EMT in pancreatic cancer cells **A.** Monolayer morphology, wound healing assay and cell invasion assay of PANC-1-A cells after transfection of ZEB1-siRNA. Quantification of cell invasion assay is shown in histogram. **B.** qRT-PCR and corresponding western blot analysis of E-cadherin, N-cadherin, and MMP-2 after inhibition of ZEB1. **C.** Monolayer morphology, wound healing assay and cell invasion assay of PANC-1-A cells after co-transfection of miR-652 mimics and pCDNA3Flag ZEB1 respectively. **D.** qRT-PCR and corresponding western blot analysis of E-cadherin, N-cadherin, and MMP-2 after co-transfection of miR-652 mimics and pCDNA3Flag ZEB1 respectively. The graphs represent data from three separate experiments. Values are significant at **P < 0.01 and *P < 0.05 as indicated.

After that, we further identified whether ZEB1 took the central roles of extracellular acidity-induced EMT regulated by miR-652. As expected, after knockdown of ZEB1, PANC-1-A cells displayed MET phenotypes in PANC-1-A cells similar to that after restoration of miR-652, evidenced by epithelial-like morphological changes as well as significant decrease in cell motility and invasion ability (Fig. 4A). In addition, there was an increased E-cadherin expression, as well as reduced expression of N-cadherin and MMP-2 (Fig. 4B) (similar in BxPC-3 cells shown in Fig. S5A and S5B).

### Re-expression of ZEB1 rescues the EMT profile of PANC-1-A cells treated with miR-652 restoration

We next explored whether the reversed EMT profile of PANC-1-A cells treated with miR-652 could be rescued by the overexpression of ZEB1. Compared to the PANC-1-A cells with miR-652 restoration, the re-expression of ZEB1 rescued the EMT transition in PANC-1-A cells (Fig. 4C and 4D) (similar in BxPC-3 cells shown in Fig. S5A and S5B). These data further confirmed that the responsively downregulated miR-652 in extracellular acidity promoted EMT by directly upregulating ZEB1.

### Both restoration of MiR-652 and reduction of ZEB-1 suppressed tumor growth and liver metastasis *in vivo*

**Figure 5:**
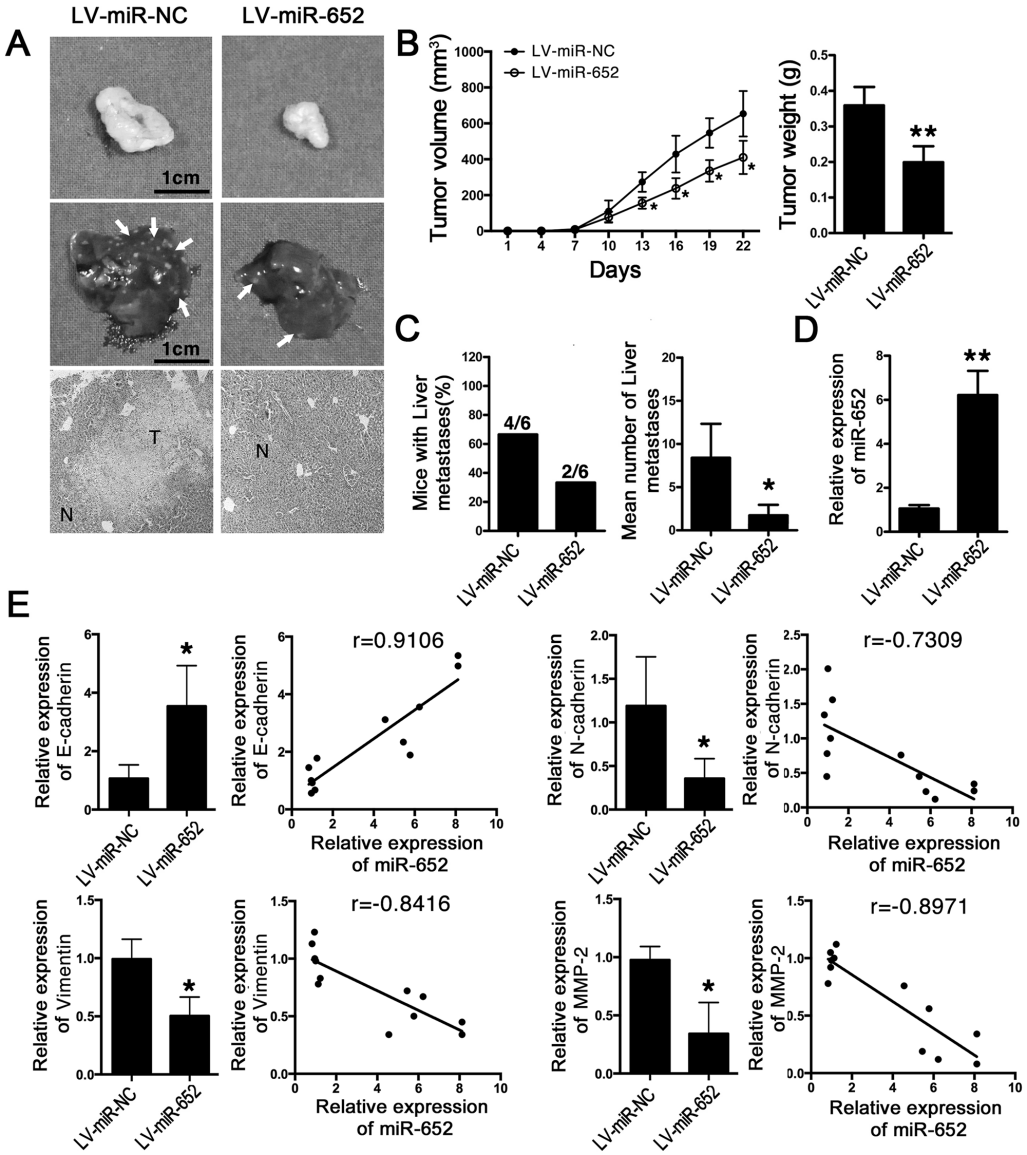
MiR-652 suppressed tumor progression and liver metastasis *in vivo* **A.** Primary tumor, metastatic nodules on liver surface (arrows represent the typical liver metastasis) and corresponding HE staining (N: normal tissue, T: metastatic tumor nodules) of mice injected with LV-miR-NC or LV-miR-652. **B.** Tumor volume and weight in LV-miR-NC and LV-miR-652 groups. **C.** The ratio of mice with liver metastasis was calculated (*n* = 6 mice per group) and the number of metastatic nodules on liver surface was counted. **D.** qRT-PCR showed miR-652 expression in primary tumors. U6 was used as the endogenous control for miR-652. **E.** The mRNA levels of epithelial marker (E-cadherin) and mesenchymal markers (N-cadherin, Vimentin, MMP-2). The correlations between miR-652 and E-cadherin/N-cadherin/Vimentin/MMP-2 in nude mice tumor samples were also measured. Values are significant at **P < 0.01 and *P < 0.05 as indicated.

We further investigate the effects of miR-652 and ZEB1 on tumor progression *in vivo*. Firstly, we transfected LV-miR-652 and LV-ZEB1-siRNA into PANC-1 cells and identified their proliferative inhibited effects (Fig. S6). After 21 days, compared with LV-miR-NC group, primary tumor growth and volume of mice injected with LV-miR-652 PANC-1 cells were significantly suppressed (Fig. 5A upper and 5B). Meanwhile, compared with LV-miR-NC group, evidenced decrease in the number of tumor metastasis was observed in LV-miR-652 group (Fig. 5A middle and 5C right). HE staining of liver sections also displayed LV-miR-652 group had much fewer metastases than that in control group (Fig. 5A lower). Moreover, we explored the effects of miR-652 on liver metastasis, which tightly correlated with EMT. Compared with 4 mice with metastasis in NC group, metastasis was only occurred in 2 mice with fewer metastatic nodules (Fig. 5C left).

Furthermore, to verify the antitumor effects caused by miR-652 treatment, we detected the expression of miR-652 in subcutaneous xenografts of pancreatic cancer by qRT-PCR. The qRT-PCR results demonstrated that miR-652 expression was sharply increased in LV-miR-652 group compared with LV-miR-NC group (*p* < 0.05) (Fig. 5D). Furthermore, the correlations between miR-652 and E-cadherin (*r* = 0.9106)/N-cadherin (*r* = −0.7309)/Vimentin (*r* = −0.8416)/MMP-2 (*r* = −0.8971) in primary tumor mass were further identified (Fig. 5E).

Similar results were observed in PANC-1 cells transfected with LV-ZEB1-siRNA (Fig. S7). At last, ZEB1 expression in tumors and liver metastases were identified by IHC and results showed that ZEB1 was remarkably reduced in tumors of both LV-miR-652 and LV-ZEB1-siRNA groups. However, there was no significant difference of ZEB1 expression in liver metastases among LV-miR-652, LV-ZEB1-siRNA and corresponding NC groups (Fig. S8).

### MiR-652 was significantly downregulated and antagonistically associated with overexpressed ZEB1 in pancreatic cancer tissues

**Figure 6:**
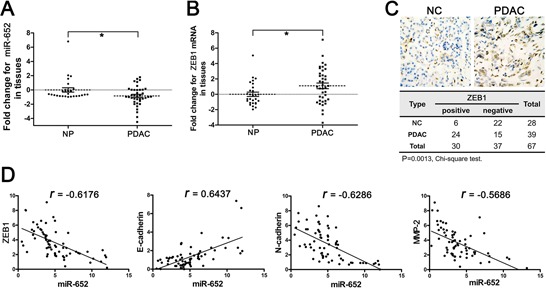
Deregulated miR-652-ZEB1 pathway in pancreatic cancer tissues **A.** The relative fold changes of miR-652 measured by qRT-PCR in NP and PDAC tissues. **B.** The relative fold changes of ZEB1 measured by qRT-PCR in NP and PDAC tissues. **C.** (*Upper*) immunohistochemical analysis of ZEB1 in representative tissues from NP and PDAC group (X400). (*Lower*) Chi-square analysis showed the expression of ZEB1 measured by immunohistochemistry. **D.** miR-652 positively correlated with E-cadherin (*r* = 0.6437), inversely correlates with ZEB1 (*r* = −0.6176), N-cadherin (−0.6286) and MMP-2 (*r* = −0.5686). U6 was used as the endogenous control for miR-652 and GAPDH for ZEB1, E-cadherin, N-cadherin and MMP-2. Values are significant at **P < 0.01 and *P < 0.05 as indicated.

We next analyzed the expression of miR-652 and ZEB1 in PDAC (pancreatic ductal adenocarcinoma) and normal pancreatic tissues to further identify its roles in development of pancreatic cancer. As shown in Fig. 6A, miR-652 expression was significantly decreased in PDAC tissues compared to normal pancreas tissues. In addition, the clinicopathological characteristics of pancreatic cancer patients demonstrated that lower miR-652 expression correlated with progressive stage, lymphatic invasion, vascular infiltration and distant metastasis (Table 2). Moreover, compared to normal pancreas tissues, tissues of PDAC showed an obviously elevated ZEB1 mRNA expression (Fig. 6B). Accordingly, IHC results showed ZEB1 protein was significantly upregulated in PDAC tissues compared to normal pancreas tissues (21.4% vs. 61.5%; *p* = 0.0013) (Fig. 6C). In addition, we found that miR-652 was positively correlated with E-cadherin expression in tissue samples (*r* = 0.6437), while negatively correlated with ZEB1 (*r* = −0.6176), N-cadherin (*r* = −0.6286), and MMP-2 (*r* = −0.5686) (Fig. 6D). Furthermore, ZEB1 expression was found to be significantly higher in pancreatic cancer tissues classified as Stage III or IV than Stage I or II (85.7 vs. 38.9%; *p* = 0.0024). Additionally, PDAC with positive lymphatic invasion (82.4 vs. 50.0%; *p* = 0.0367) and vascular infiltration (78.3 vs. 43.8%; *p* = 0.0271) had a significant higher ZEB1 expression. However, there was no significant correlation between ZEB1 and distant metastasis (78.9 vs. 50.0%; *p* = 0.0596), although the promotional trend was observed (Table 3). Therefore, these clinical data further verified that the miR-652 might function as inhibitor in tumorigenesis of pancreatic cancer by directly targeting ZEB1.

**Table 2:** Relationship between miR-652 expression and clinicopathological factors in PC patients

Parameters	miR-652		*P*_1_*	Mean Fold change ± SEM	*P*_2_*
	high	low			
**Stage**			0.0160*		0.007*
Stage I or II (*n* = 18)	9	9		−0.213 ± 0.265	
Stage III or IV (*n* = 21)	3	18		−1.379 ± 0.300	
**Lymphatic invasion**			0.3892		0.023*
Positive (*n* = 17)	4	13		−1.404 ± 0.345	
Negative (*n* = 22)	8	14		−0.406 ± 0.257	
**Vascular infiltration**			0.0300*		0.026*
Positive (*n* = 23)	4	19		−1.245 ± 0.241	
Negative (*n* = 16)	8	8		−0.260 ± 0.376	
**Distant metastasis**			0.0482*		0.015*
Positive (*n* = 19)	3	16		−1.382 ± 0.293	
Negative (*n* = 20)	9	11		−0.327 ± 0.291	

* *P* was calculated by Chi-square test.

*P* value <0.05 was considered statistically significant.

**Table 3:** Relationship between ZEB1 expression and clinicopathological factors in PC patients

Parameters	ZEB1		*P*_1_*	Mean Fold change ± SEM	*P*_2_*
	high	low			
**Stage**			0.0024*		0.0036*
Stage I or II (*n* = 18)	7	11		0.003 ± 0.506	
Stage III or IV (*n* = 21)	18	3		2.004 ± 0.427	
**Lymphatic invasion**			0.0367*		0.0017*
Positive (*n* = 17)	14	13		2.339 ± 0.518	
Negative (*n* = 22)	11	11		0.147 ± 0.406	
**Vascular infiltration**			0.0271*		0.0046*
Positive (*n* = 23)	18	5		1.930 ± 0.445	
Negative (*n* = 16)	7	9		−0.088 ± 0.484	
**Distant metastasis**			0.0596		0.0540
Positive (*n* = 19)	15	4		1.816 ± 0.533	
Negative (*n* = 20)	10	10		0.424 ± 0.457	

* *P* was calculated by Chi-square test.

*P* value < 0.05 was considered statistically significant.

## DISCUSSION

Previous studies revealed that acidic microenvironment increased invasion potential of murine and human cancer cells, while the exact mechanism has not been defined [2, 32, 33]. The present study showed that pancreatic cancer cells demonstrated dramatic EMT phenotypes in response to the extracellular acidity demonstrating as significant increase in cell motility, invasion ability and colony formation ability. Therefore, our present research provided the evidences that the extracellular acidity might be one of the pivotal stimulator for the extreme malignant activities of pancreatic cancer cells by promoting EMT, which provides a novel insight into the interaction between the microenvironment and tumorigenesis of pancreatic cancer. Similarly, the results from Suzuki et al showed that acidic microenvironment promoted EMT in lung carcinoma cells, as well as Peppicelli et al also demonstrated that acidic micronenvironment contributed EMT of melanoma cells [20, 21].

It is of importance to study whether EMT induction in pancreatic cancer cells results from an induction or selection phenomenon in acidic microenvironment. Therefore, we compared the viability, proliferation and cell cycle between pH = 7.4 and pH = 6.4. The results showed that the acidity inhibited the proliferation of pancreatic cancer cells, as well as induced G1 arresting and we observed moderate growth of pancreatic cancer cells under acidic treatment instead of obviously cell-killing effects. These results implied that the pancreatic cancer cells had the capability of resisting in acidity and the acidity did not demonstrated obvious cytotoxity on pancreatic cancer cells. Therefore, we consider that the acidity-induced EMT is the result from induction but not selection phenomenon. Moreover, we performed soft agar colony formation assay to identify the anchorage-independent growth capacity of pancreatic cancer cells in our research. Anchorage-independent growth used to characterize cells that do not require a solid substratum for growth, such cells could be grown in suspension or soft media *in vitro* and obtained promoted metastasis ability *in vivo*. Very interestingly, the results showed that the quantity of colony formation of acidity-treated cells was more than that of normal cells, while the diameter of the clone in acidity-treated cells was smaller. Therefore, our results indicated that the acidity increased colony formation by enhancing anchorage-independent growth ability, in spite of restraining the proliferation of pancreatic cancer cells.

Accumulative evidences illustrated that ZEB1 plays critical roles in EMT of cancers including pancreatic cancer [30, 31]. The present study displayed that the remarkable increase of ZEB1 was observed in pancreatic cancer cells treated with acidity, while knockdown of ZEB1 induced MET-like transformation in acidity treated pancreatic cancer cells. This result suggested that ZEB1 might be the critical regulator for the aicidity-induced EMT in pancreatic cancer cells. Since miRNAs acting as the crucial stress-responsive mediator by involving in the signal transduction in response to environmental stimulus [28, 29], we hypothesized that acidic stimulus might lead to miRNAs alteration and further contribute to EMT by upregulating ZEB1. According to the data of miRNAs-microarray and qRT-PCR validation in pancreatic cancer cells, the downregulated miR-652, predicted as the potential regulator for ZEB1 expression, was found to be one of the most attractive candidates functioning in acidic microenvironment. MiR-652 was reported to be noticeably downregulated in esophageal cancer patients with poor prognosis, and Sukata et al. also reported circulating miR-652 might act as a possible indicator of rat hepatocarcinogenesis from early stages [34, 35]. After that, we detected whether miR-652 exerts critical roles in acidity-induced EMT of pancreatic cancer cells. After restoration of miR-652, the acidity treated pancreatic cancer cells showed MET-like transformation, which was rescued by re-expression of ZEB1. On the other hand, inhibition of miR-652 induced EMT process in normal parental pancreatic cancer cells. In addition, the results *in vivo* further demonstrated that lower tumor proliferation and dramatic less liver metastasis were appeared both in miR-652 overexpression and ZEB1 knockdown xenotransplantation models. Meanwhile, the IHC results validated ZEB1 expression was significantly decreased in the primary tumors, but not in the liver metastasis of LV-miR-652 and LV-ZEB1-siRNA groups. It suggested those pancreatic cancer cells with lower ZEB1 expressing would lose the ability to metastasis and only those cells with higher ZEB1 expression could form liver metastasis, which might be the reason for the discrepancy of ZEB1 expression in the primary tumor and liver metastasis.

To further identify the downregulated miR-652 and increased ZEB1 contributing to metastasis in pancreatic cancer, we detected the miR-652 and ZEB1 expression in pancreatic cancer and normal pancreas tissue samples. The results showed miR-652 was significantly decreased in pancreatic cancer tissues and negatively correlated with the upregulated ZEB1 expression. In addition, miR-652 acted as suppressors of EMT in pancreatic tissues, evidenced by the expression of miR-652 was significantly positively correlated with E-cadherin while negatively with N-cadherin in tissue samples. At last, we found the decreased miR-652 and increased ZEB1 were also correlated to late tumor stage, lymphatic invasion and vascular infiltration.

In summary, our current findings implicated an extracellular acidic microenvironment-miR-652/ZEB1-EMT axis which might involve in tumorigenesis of pancreatic cancer. Furthermore, miR-652 might serve as novel targets and prognostic markers for pancreatic cancer. Moreover, it might provide a new insight into the correlation between microenvironment and tumor progression.

## MATERIALS AND METHODS

### Clinical samples

All the samples were collected from Pancreatic Disease Institute of Union Hospital (Wuhan, China) from September 2011 to October 2013. The patients included 41 male and 26 female with average age 57.2 years (range: 39~73). 39 samples were from patients with pancreatic ductal adenocarcinoma (PDAC) who had not received any radiotherapy or chemotherapy before excision or biopsy. Those patients were treated with pancreatectomy or palliative surgery including implantation of I^125^ seeds as well as choledochojejunostomy and gastroenterostomy, depending on the National Comprehensive Cancer Network (NCCN) guideline for pancreatic cancer (version 1. 2011). 28 normal pancreas (NP) samples were taken from peripheral tissues away from tissue of serous cystadenoma or insulinoma. The original histopathological reports were obtained from each case and the diagnosis of pancreatic cancer was confirmed. Immediately after surgical removal, tissue samples were either snap-frozen in liquid nitrogen or fixed in 10% buffered formalin solution and then embedded in paraffin (for histological analysis). The complete details of the entire study design and procedures involved were in accordance with the Declaration of Helsinki. All participants and their parents gave their written informed consent to participate in the study after the risks and benefits we discussed in detail. This study was approved by the ethics committee of the Union Hospital of Huazhong University of Science and Technology.

### Cell culture

The human pancreatic cancer cell lines AsPC-1, BxPC-3, PANC-1, SW1990 and MIAPaCa-2 were routinely cultured at 37°C in RPMI 1640 medium supplemented with 10% fetal bovine serum, penicillin (100U/ml) and streptomycin (100 μg/ml) in an incubator with 95% air and 5% CO_2_. To vary the medium’ pH, we added 20 mM 2-(N-morpholino)-ethane-sulfonic acid and 20 mM Tris-(hydeoxymethyl)-aminomethane [36].

### Immunohistochemistry

Immunohistochemical staining was done on paraffin-embedded tissue. Briefly, paraffin sections of 3-μm thick were cut and probed. After rehydration, samples were treated with solution containing 0.3% hydrogen peroxide for 30 min to block endogenous peroxidase activity. After antigen retrieval in citrate buffer, the sections were incubated with the primary antibody (1:100 to 1:150in PBS, overnight at 4°C, ZEB1 (ab180905) were from Abcam. As negative control, sections were incubated with PBS instead of the primary antibody. This was followed by incubation with biontinylated secondary antibody (1:200; Santa Cruz Biotechnology, Santa Cruz, CA) for 30 min at 37°C. The signal was amplified by avidin-biotin complex formation and developed with DAB followed by counterstaining with hematoxylin, dehydrated in alcohol and xylene, mounted, and analyzed by standard light microscopy. Two investigators simultaneously assessed the results of immunostaining without knowledge of the patient clinicopathological details. Immunohistochemical staining of ZEB1 was estimated as positive expression when cases with ≥30% positive tumor cells in a section.

### MiRNAs expression analysis

MiRNA expression profiling was performed according to the manufacturer's instruction. Hybridization stared from 2.5 μg total RNA according to Ribo miRNA Hybridization Protocol and array version was RiboArray™ mi*DETECT*™ Human Array 1 × 12K. Standard selection criteria to identify differentially expressed miRNAs are established at *P* < 0.05 and fold difference > 2. PANC-1 cell line was utilized for miRNAs expression profiling in our study.

### RNAs isolation and quantitative real-time PCR

Total RNA was extracted from the patients’ cancer samples or the culture cells using TRIzol (Invitrogen) according to the manufacturer's protocol. MRNAs and miRNAs were reverse transcribed according to the protocol of Prime Script RT Master Mix (TaKaRa) and One Step Prime Script miRNA cDNA Synthesis Kit (TaKaRa), respectively. QRT-PCR for both mRNAs and miRNAs were performed as described in the method of SYBER premix Ex Taq (TaKaRa). The reactions were monitored using a preheated real-time instrument (ABI StepOne plus Real-Time PCR System). The PCR conditions were 30s at 95°C, followed by 40 cycles of 95°C for 5s, 60°C for 30s. Real-time PCR was done in triplicate, including no-template controls. The expression of mRNAs and miRNAs were defined from the threshold cycle (Ct), and relative expression of mRNAs and miRNAs was calculated using the comparative CT (2^−ΔΔCT^) method. The expression of mRNA and miRNA in tissues or cultured cells was normalized with reference to GAPDH and U6 small nuclear RNA respectively. Primers of qRT-PCR were listed in Supplementary Table 1.

### MicroRNAs and RNAs transfection

Stability-enhanced miR-652 precursor (miR-652 mimics), miR-652 inhibitor (miR-652 inhibitor) and their matched NC (mimics control and inhibitor control), as well as a short interference RNA (siRNA) for human ZEB1 (ZEB1-siRNA) was from Invitrogen (Shanghai, China). Control siRNA oligonucleotide (siRNA control), which does not match any known human coding cDNA, was also designed and used from Invitrogen (Shanghai, China). BxPC-3 and PANC-1 cells were grown to 50% confluence in 12-well plates, and transfected with the miRNAs or siRNAs in Opti-MEM media (Invitrogen) using LipofectamineTM 2000 (Invitrogen) according to the manufacturer's instructions. A final concentration of 50 nM microRNA mimics, microRNA inhibitor, siZEB1 and their matched control was used. Plasmids pCDNA3FlagZEB1 and control vector were from RiboBio (Guangzhou, China). The plasmids were transfected into cells according to the manufacturer's protocol. The effect of the transient transfection was confirmed by qRT-PCR and western blot analysis. Lentiviral miR-652 (LV-miR-652), lentiviral ZEB1-siRNA (LV-ZEB1-siRNA) and corresponding control lentiviral vector (LV-miR-NC and LV-siRNA-NC) were constructed by Genechem Company (Shanghai, China), and transduced to the PANC-1 cells according to the manufacturer's instructions [37].

### Luciferase reporter assays

PANC-1 cells were seeded in 24-well plates (70%–80% confluence) and transfected with pmiR-RB-Report™-ZEB1 (wild type ZEB1 3′UTR, WT) and pmiR-RB-Report™–Control (mutant type ZEB1 3′UTR, MUT) for miR-652 (RiboBio, Guangzhou, China) respectively were purchased from with Lipofectamine 2000 (Invitrogen). Cells were co-transfected with 50 nM miR-652 or 50 nM miR-NC respectively. Luciferase activity was determined using the dual luciferase assay system (Promega; Madison, WI) after 48 h of transfection. Luciferase activity was normalized to Renilla luciferase activity.

### Western blot analysis

Whole cell lysates were prepared in lysis buffer (50 mM Tis-HCl, 150 mM NaCl, 0.5% sodium deoxycholate, 0.1%SDS, 1% NP-40) and supernatants collected by centrifugation. Equal amounts of protein were denatured in SDS sample buffer and separated on 8%, 10% polyacrylamide gels according to the molecular weight of the target proteins. Separated proteins were transferred to PVDF membrane. The membranes were blocked with 5% non-fat milk TBST (TBS containing 0.05% Tween 20), incubated with primary antibodies, and subsequently with alkaline phosphatase-conjugated secondary antibody. Protein expression was detected by chemiluminescence (ECL, Amersham). Antibodies against E-cadherin, β-catenin, Vimentin, MMP-2, GAPDH were purchased from Cell Signaling (Danvers, MA). Antibodies against N-cadherin, ZEB1 were from Santa Cruz Biotechnology (Santa Cruz, CA). Expression of GAPDH was used as a loading control. Band density analysis was performed in Adobe Photoshop CS5.

### Cell invasion assay

Invasion assays were performed in triplicate using Transwell chambers for 24-well plates (0.8-μm pore size; Corning, Costar) coated with ECM gel (40 μl; Sigma) according to the manufacturer's instructions. Cells (2 × 10^4^ to 6 × 10^4^) were plated in 200 μl RPMI 1640 medium with 0.1% fetal bovine serum into the upper chamber. The lower chamber was filled with 700 μl RPMI 1640 medium with 30% fetal bovine serum. After culture for 24 h to 72 h, noninvaded cells were mechanically removed by a cotton swab. The invaded cells at underside of the membrane were fixed with 4% formalin and stained with 0.1% crystal violet for visualization. Cells were counted in ten respective microscopic fields (× 40 magnification) and photographed.

### Wound healing assay

“Wound healing” assay was used to detect the alteration of cell motility. Cells were initially seeded uniformly onto 12-well culture plates with an artificial “wound” carefully created at 0 h, using a P-200 pipette tip to scratch on the confluent cell monolayer, microphotographs were taken at 0 and 48 h (×20 magnification).

### Soft agar colony formation assay

Cells (1250cells/well) were suspended in 10% FBS-RPMI 1640 containing 0.3% agar. The cells were then placed into a 24-well culture plate containing a hard agar base composed of 10% FBS-RPMI 1640 and 0.5% agar. The cultures were returned to the incubator and fed every 2 days with 250 μl of acidic medium and normal medium respectively in two groups. The plates were incubated for 2 weeks. The cells were stained with 0.05% crystal violet overnight at 37 degree. Colonies were visualized and counted by light microscopy at X20 magnification. Soft agar assay were performed in triplicate.

### Animal experiments

All animal protocols were approved by the Institutional Animal Care and Treatment Committee of Tongji Medical College of Huazhong University of Science and Technology (Wuhan, Hubei, China). LV-miR-652, LV-ZEB1-siRNA, LV-miR-NC and LV-siRNA-NC transfected PANC-1 cells were harvested by trypsinization and resuspended in RPMI 1640. The cells (8 × 10^6^) in 0.1ml serum-free PBS were injected subcutaneously (SC) into the right flank of 3-week-old female BALB/c nu/nu mice (*n* = 6 mice per group). The tumor volume was measured with a caliper every 3 days and calculated according to the formula: Tumor volume= length × width^2^/2. Animals were sacrificed 3 weeks after injection, and solid tumor tissues were removed and weighed. Tumor metastasis on liver surfaces were counted under a dissecting microscope and photographed, subsequently the liver tissues were paraffin embedded and sectioned for further pathologic analysis.

### Statistical analysis

Results were expressed as means ± standard deviation (SD). Standard selection criteria of the miRNAs profiling to identify differentially expressed miRNAs are established at *P* < 0.05 and fold difference > 2. Group's comparisons were analyzed with variance (ANOVA) or Student's *t* test. The expression of miR-652 and ZEB1 in PDAC and normal pancreas tissues was obeyed normal distribution and were further analyzed with *t*-test. The relationships between the expression of miR-652/ZEB1 and clinical characteristics of PDAC tissue were analyzed using χ2 tests. Correlation analysis was performed using Spearman's correlation analysis. *P*-values less than 0.05 were considered to be significant.

## SUPPLEMENTARY FIGURES AND TABLE


