# Supplementary material for: MiR-652 inhibits acidic microenvironment-induced epithelial-mesenchymal transition of pancreatic cancer cells by targeting ZEB1
Source: Oncotarget. 2015 Oct 19;6(37):39661–75. doi: 10.18632/oncotarget.5350 (PMC4741853; doi:10.18632/oncotarget.5350)
Supplement: Supplementary file 1 [file oncotarget-06-39661-s001.pdf]

SUPPLEMENTARY FIGURES AND TABLE

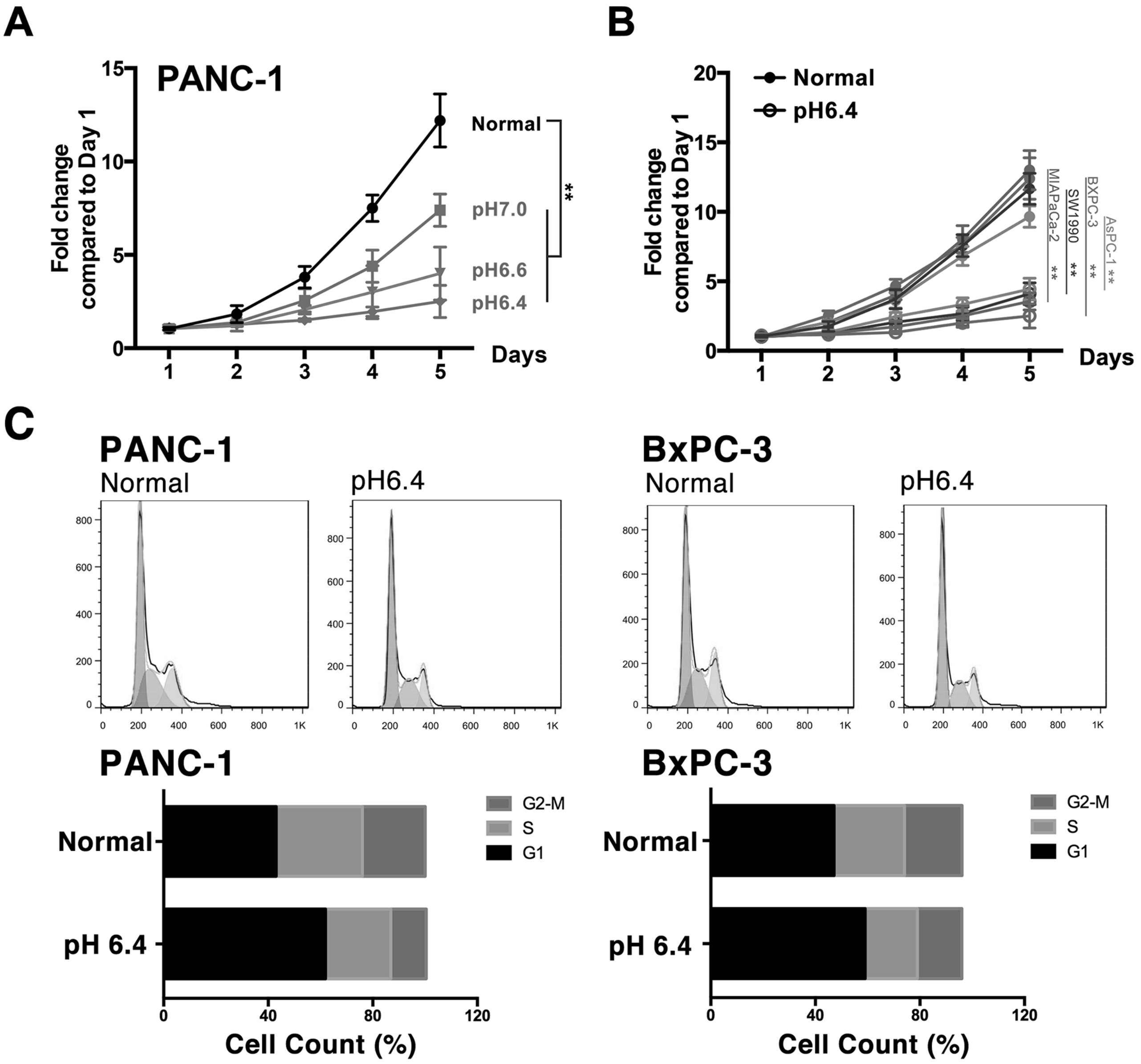

Supplementary Figure S1: Acidic environment leaded to proliferative inhibition of pancreatic cancer cell lines. A. Proliferative rate of PANC-1 cells that cultured in normal (pH7.4) and acidic medium (pH7.0, pH6.6, pH6.4) were measured by MTT assay. **B.** MTT was used to identify the proliferative rate of AsPC-1, BxPC-3, SW1990 and MIAPaCa-2 cultured in normal (7.4) and acidic medium (6.4). **C.** Acidity leaded to remarkable G1-phase arrest in pancreatic cancer cells (data of PANC-1 and BxPC-3 cell lines were shown). The graphs represent data from three separate experiments. Values are significant at **, $P < 0.01$ and *, $P < 0.05$ as indicated.

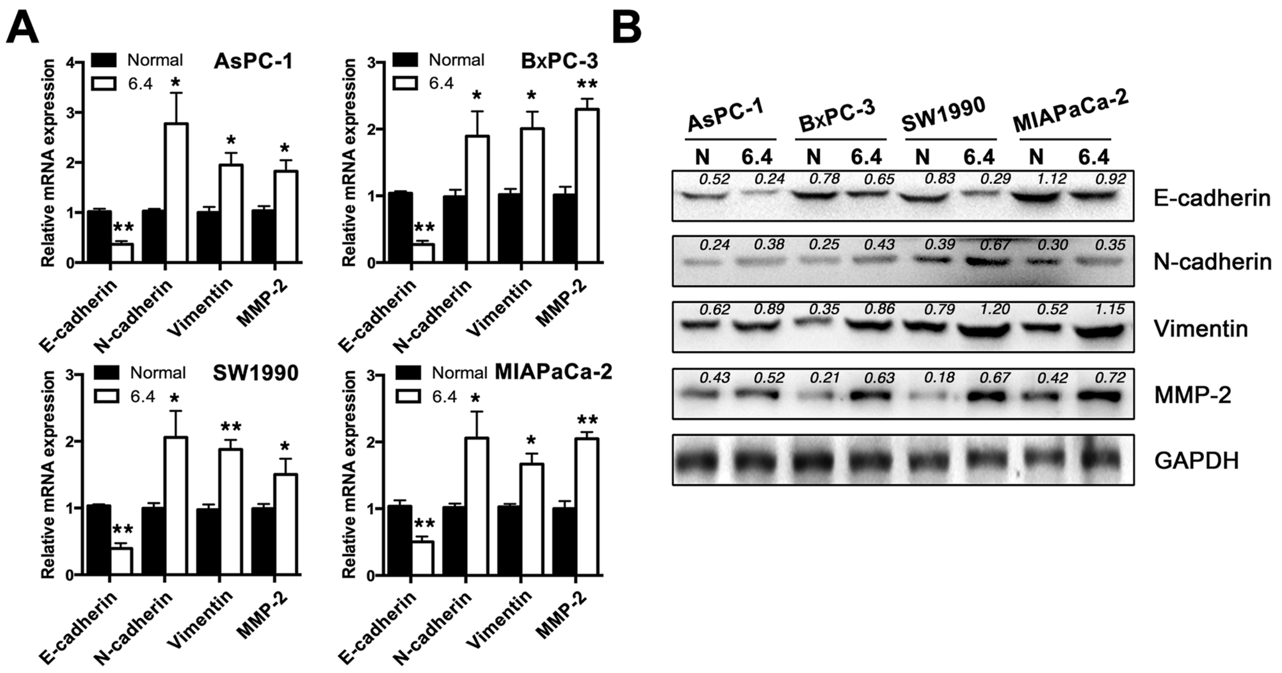

Supplementary Figure S2: Acidity potentiated EMT in pancreatic cancer cell lines. A. QRT-PCR analysis of epithelial markers (E-cadherin), mesenchymal markers (N-cadherin, Vimentin and MMP-2) protein levels in AsPC-1, BxPC-3, SW1990 and MIAPaCa-2 kept at normal (pH7.4) and acidic medium (pH6.4) as indicated up to 72 h. **B.** Western blot was performed to measure the protein level of E-cadherin, N-cadherin, Vimentin and MMP-2 in pancreatic cancer cell lines cultured in normal (pH7.4) and acidic medium (pH6.4) up to 72 h. The graphs represent data from three separate experiments. Values are significant at $^{**}P < 0.01$ and $^{*}P < 0.05$ as indicated.

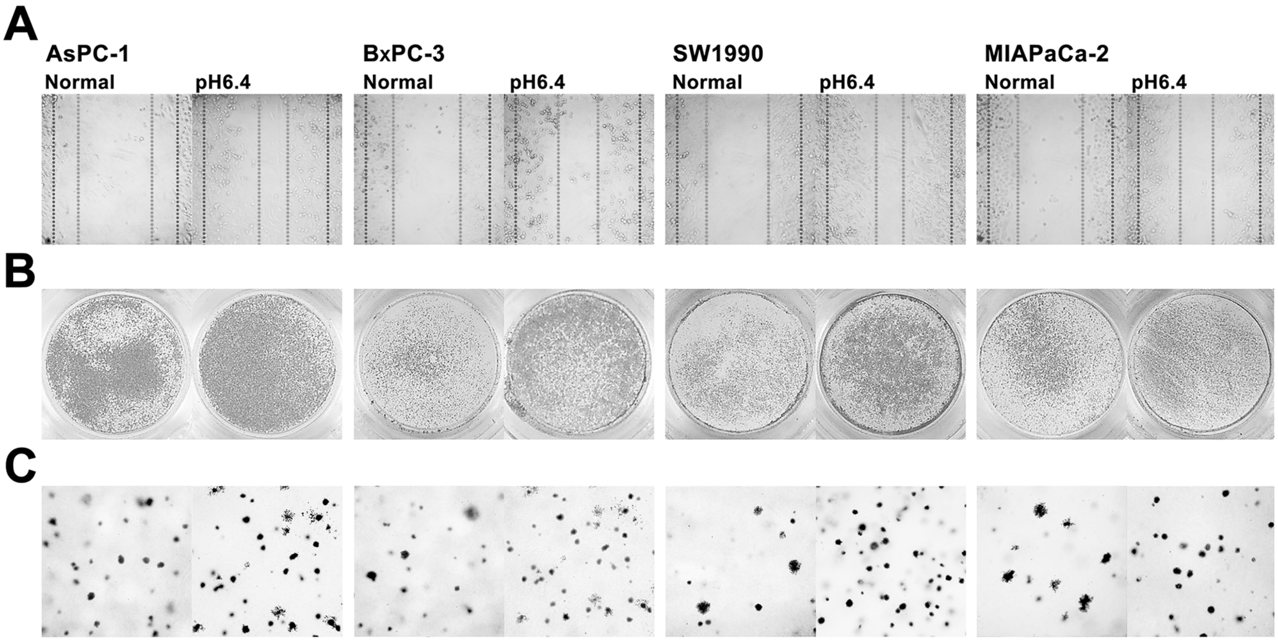

Supplementary Figure S3: Acidity resulted in promoted motility, invasion and colony formation capacities in pancreatic cancer cell lines. **A.** Acidic pH increased cell motility (wound healing assay, X20). **B.** Acidic medium leaded to promoted invasion ability (cell invasion assay). **C.** Acidity resulted in enhanced colony formation ability (soft agar colony formation assay, X20).

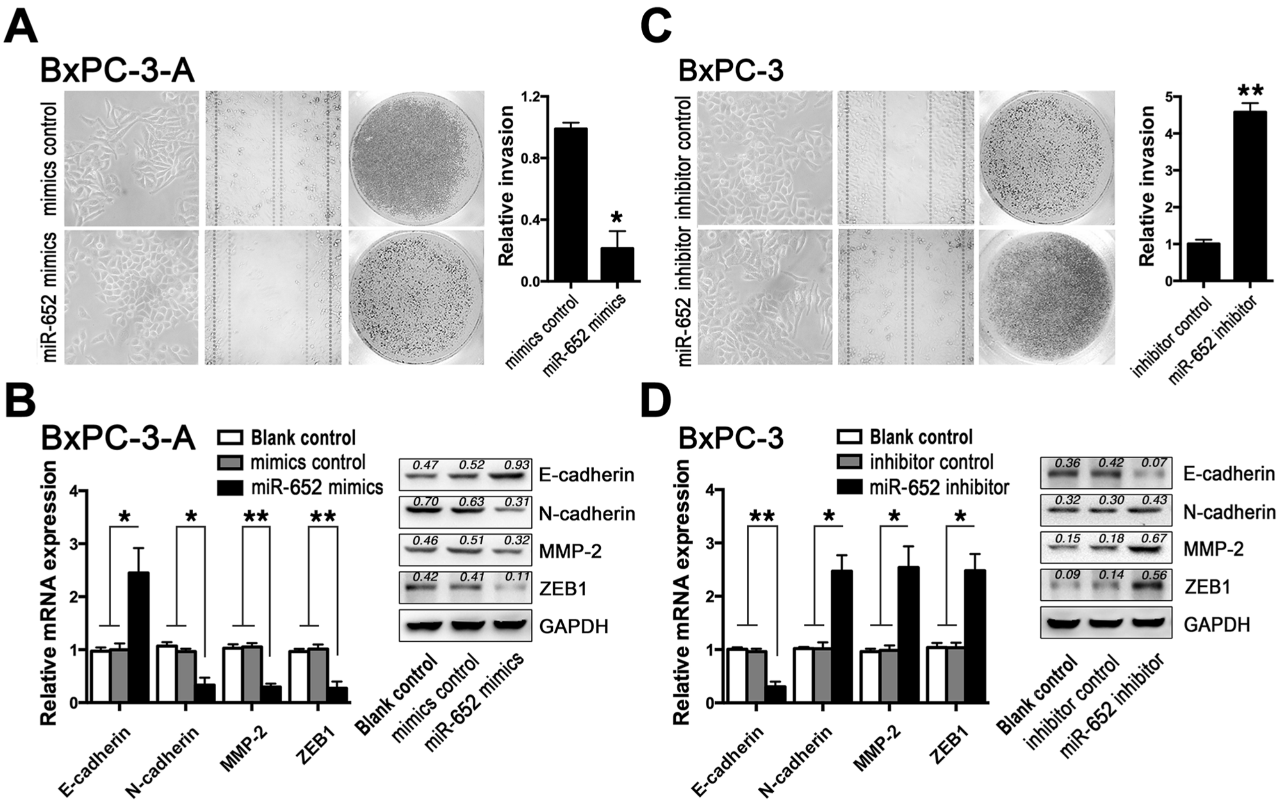

Supplementary Figure S4: ZEB1 mediated miR-652 function in acidity-induced EMT. A. Monolayer morphology, wound healing assay and cell invasion assay of BxPC-3-A (BxPC-3 incubated in pH 6.4 value medium for 72 h) after transfection of miR-652 mimics. Quantification of cell invasion assay is shown in histogram. **B.** qRT-PCR and corresponding western blot analysis of E-cadherin, N-cadherin, MMP-2 and ZEB1 in BxPC-3-A after transfection of miR-652 mimics. **C.** Monolayer morphology, wound healing assay and cell invasion assay of BxPC-3 cells after transfection of miR-652 inhibitor. Quantification of cell invasion assay is shown in histogram. **D.** qRT-PCR and corresponding western blot analysis of E-cadherin, N-cadherin, MMP-2 and ZEB1 in BxPC-3 after transfection of miR-652 inhibitor. The graphs represent data from three separate experiments. Values are significant at **, *P* < 0.01 and *, *P* < 0.05 as indicated.

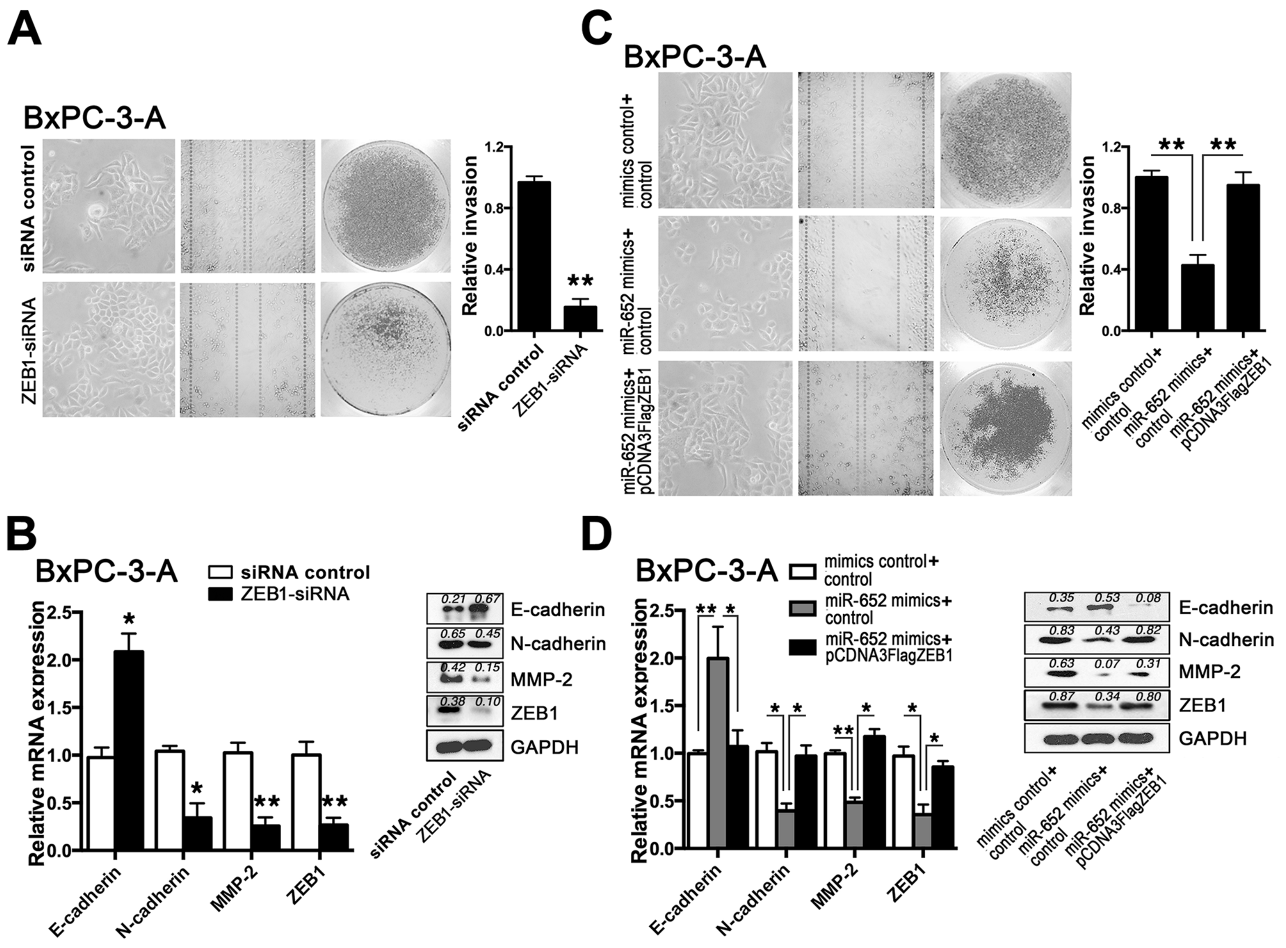

Supplementary Figure S5: Downregulation of ZEB1 reversed the extracellular acidity-induced EMT in pancreatic cancer cells. A. Monolayer morphology, wound healing assay and cell invasion assay of BxPC-3-A cells after transfection of ZEB1-siRNA. Quantification of cell invasion assay is shown in histogram. **B.** qRT-PCR and corresponding western blot analysis of E-cadherin, N-cadherin, and MMP-2 after inhibition of ZEB1. **C.** Monolayer morphology, wound healing assay and cell invasion assay of BxPC-3-A cells after co-transfection of miR-652 mimics and pCDNA3Flag ZEB1 respectively. **D.** qRT-PCR and corresponding western blot analysis of E-cadherin, N-cadherin, and MMP-2 after co-transfection of miR-652 mimics and pCCDNA3Flag ZEB1 respectively. The graphs represent data from three separate experiments. Values are significant at **, $P < 0.01$ and *, $P < 0.05$ as indicated.

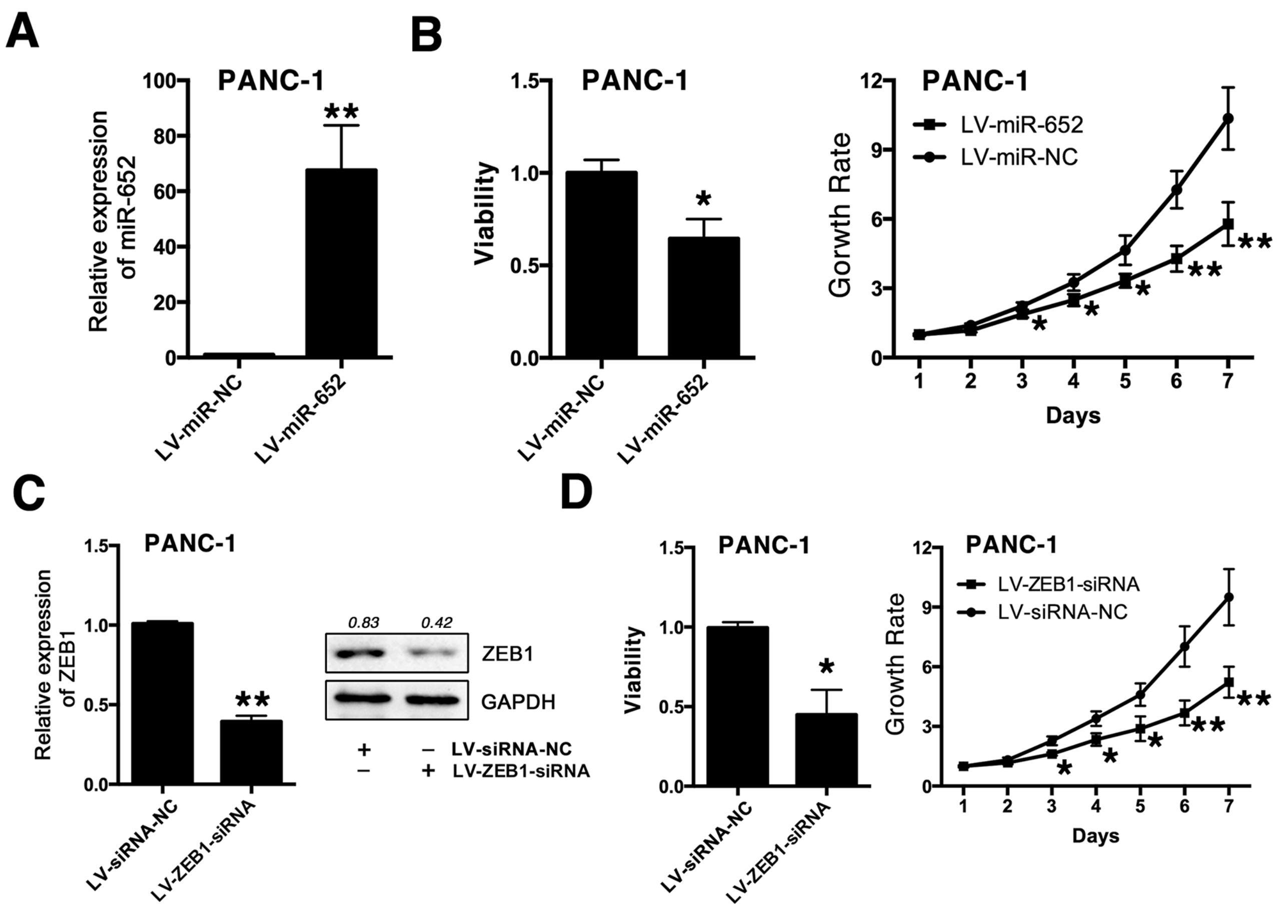

Supplementary Figure S6: LV-miR-652 and LV-ZEB1-siRNA prohibited proliferation of PANC-1 cells significantly. **A.** qRT-PCR was performed to identify transfection efficiency of LV-miR-652 in PANC-1 cells. **B.** Viability and proliferation of PANC-1 cells that was transfected with LV-miR-652 and LV-miR-NC. **C.** Transfection efficiency of LV-ZEB1-siRNA in PANC-1 cells was measured by qRT-PCR and western blot. **D.** Viability and proliferation of PANC-1 cells that was transfected with LV-ZEB1-siRNA and LV-siRNA-NC. The graphs represent data from three separate experiments. Values are significant at **, $P < 0.01$ and *, $P < 0.05$ as indicated.

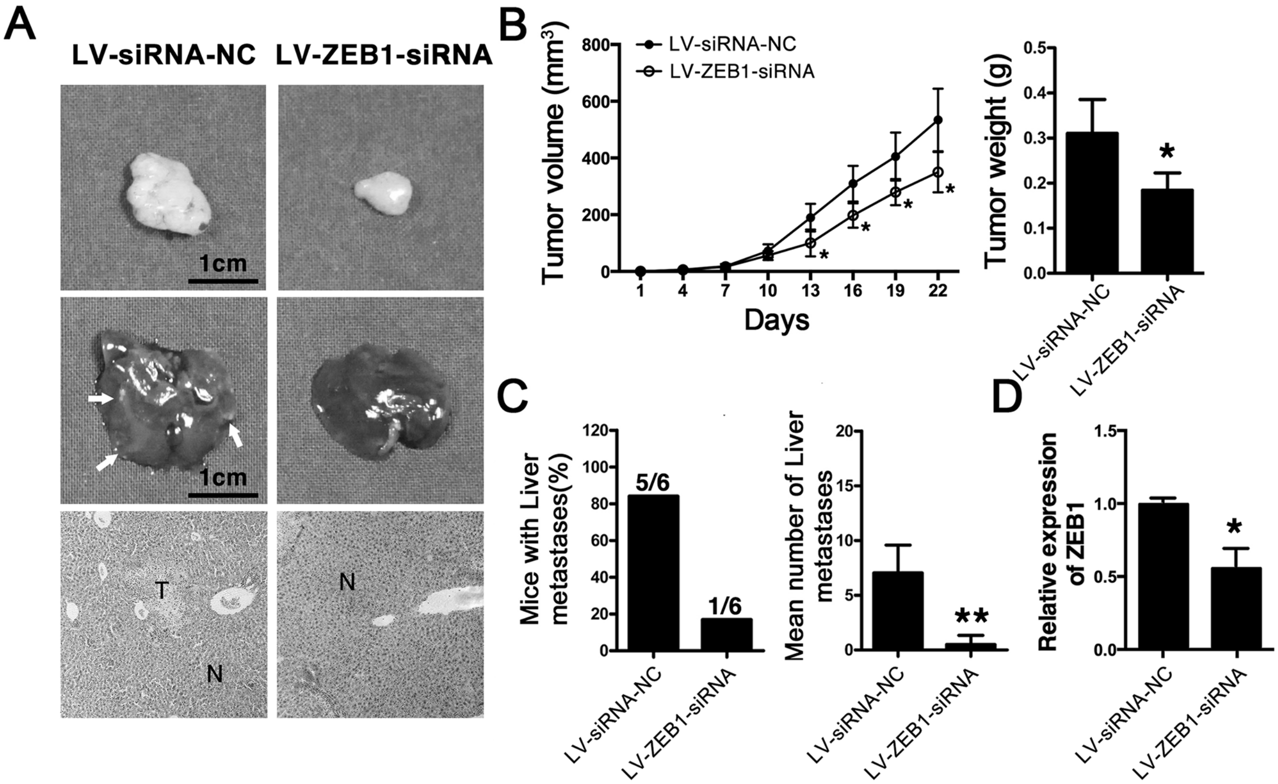

Supplementary Figure S7: ZEB1 promoted tumor progression and liver metastasis *in vivo*. A. Primary tumor, metastatic nodules on liver surface (arrows represent the typical liver metastasis) and corresponding HE staining (N: normal tissue, T: metastatic tumor nodules) of mice injected with LV-siRNA-NC or LV-ZEB1-siRNA. **B.** Tumor volume and weight in LV-siRNA-NC or LV-ZEB1-siRNA groups. **C.** The ratio of mice with liver metastasis was calculated (n = 6 mice per group) and the number of metastatic nodules on liver surface was counted. **D.** qRT-PCR showed miR-652 expression in primary tumors. U6 was used as the endogenous control for miR-652. The graphs represent data from three separate experiments. Values are significant at **, $P < 0.01$ and *, $P < 0.05$ as indicated.

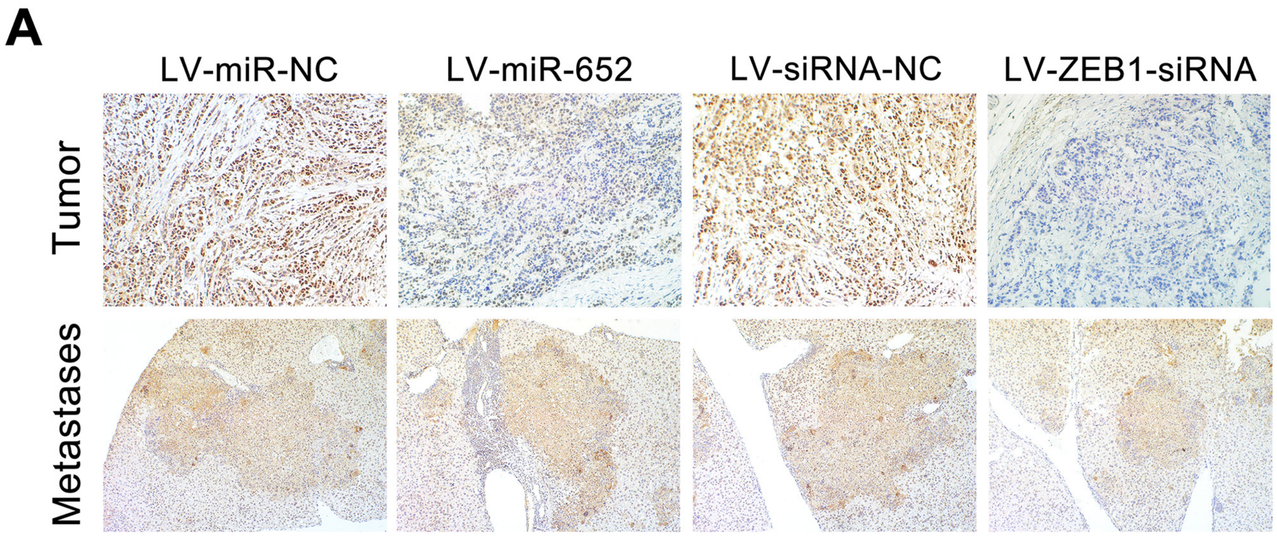

Supplementary Figure S8: Immunohistochemical analysis of ZEB1 in primary tumor and liver metastases. A. ZEB1 expression of representative primer tumor and liver metastases in LV-miR-NC, LV-miR-652, LV-siRNA-NC and LV-ZEB1-siRNA groups was measured by immunohistochemical analysis.

Supplementary Table S1: The sequence of PCR primers

Primer	Sequence
ZEB1	forward: 5′-AAGAATTCACAGTGGAGAGAAGCCA-3′
	reverse: 5′-CGTTTCTTGCAGTTTGGGCATT-3′
E-cadherin	forward: 5′-ATTCTGATTCTGCTGCTCTTG-3′
	reverse: 5′-AGTAGTCATAGTCCTGGTCTT-3′
N-cadherin	forward: 5′-GATGTTGAGGTACAGAATCGT-3′
	reverse: 5′-GGTCGGTCTGGATGGCGA-3′
Vimentin	forward: 5′-TGTCCAAATCGATGTGGATGTTTC-3′reverse: 5′-TTGTACCATTCTTCTGCCTCCTG-3′
MMP2	forward: 5′-TTGATGGCATCGCTCAGATC-3′reverse: 5′-TTGTCACGTGGCGTCACAGT-3′
GAPDH	forward: 5′-TGAACGGGAAGCTCACTGG-3′
	reverse: 5′-TCCACCACCCTGTTGCTGTA-3′
miR-652	forward: 5′-CAACCCTAGGAGAGGGTGCCATTCA-3′
U6	forward: 5′-GCTTCGGCAGCACATATACTAAAAT-3′
